# The “curved lead pathway” method to enable a single lead to reach any two intracranial targets

**DOI:** 10.1038/srep40533

**Published:** 2017-01-11

**Authors:** Chen-Yu Ding, Liang-hong Yu, Yuan-Xiang Lin, Fan Chen, Zhang-Ya Lin, De-Zhi Kang

**Affiliations:** 1 Department of Neurosurgery, The First Affiliated Hospital of Fujian Medical University, Fuzhou, China

## Abstract

Deep brain stimulation is an effective way to treat movement disorders, and a powerful research tool for exploring brain functions. This report proposes a “curved lead pathway” method for lead implantation, such that a single lead can reach in sequence to any two intracranial targets. A new type of stereotaxic system for implanting a curved lead to the brain of human/primates was designed, the auxiliary device needed for this method to be used in rat/mouse was fabricated and verified in rat, and the Excel algorithm used for automatically calculating the necessary parameters was implemented. This “curved lead pathway” method of lead implantation may complement the current method, make lead implantation for multiple targets more convenient, and expand the experimental techniques of brain function research.

Deep brain stimulation (DBS) is an effective treatment to reduce tremor notably in late-stage Parkinson’s disease^1^,^2^,^3^,^4^,^5^,^6^, as well as an important method to research the brain function^7^,^8^,^9^,^10^,^11^,^12^,^13^,^14^,^15^,^16^,^17^. It has been reported^18^,^19^,^20^,^21^,^22^,^23^,^24^,^25^,^26^,^27^,^28^,^29^,^30^,^31^,^32^,^33^,^34^,^35^,^36^ that implanting leads to multiple targets is of important practical value. In terms of treating diseases, quadruple-targeted DBS might lead to better treatment results compared to dual-targeted DBS^18^,^19^,^20^,^21^,^22^,^23^,^24^. In animal studies, there are many researchers who stimulate and/or recorde multiple nuclei simultaneously^25^,^26^,^27^,^28^,^29^,^30^,^31^,^32^,^33^.

However, it is difficult to implant a single lead into multiple targets simultaneously with the current method of lead implantation. Only limited research^34^,^35^,^36^ demonstrated that a single lead can be delivered to two targets in sequence through a “straight lead pathway”. This “straight lead pathway” method is suitable for only a few combinations of targets, such as internal globus pallidus (GPi) + external globus pallidus (GPe)^35^ or internal capsule + accumbens nucleus^36^, and is not applicable when the distance between the two targets is far, or the extension of the line that connects the two targets is not a suitable pathway for lead implantation. In most cases, the more targets researchers stimulate and/or recorde, the more leads are implanted^18^,^19^,^20^,^21^,^22^,^23^,^24^,^27^,^31^. However, the increasing number of leads could lead to increased incidence of complications^37^, and increased medical cost related to purchasing the leads. Therefore, if a single lead can be implanted into any two selected targets, it might be a beneficial addition to the current method of lead implantation. This paper proposes a “curved lead pathway” method to enable a single lead to reach two intracranial targets.

## Materials and Methods

### The theoretical position of the “curved lead pathway”

The key to enable a single lead to reach any two selected targets is the use of auxiliary devices to secure the curved pathway for lead implantation (the “curved lead pathway”). Therefore, it is necessary to determine the theoretical position of the “curved lead pathway”. First, find a plane that passes through both targets and is perpendicular to the XOY plane of the accessory (the plane of “bottom frame”). There is one and only one of such a plane, which is defined as the “lead pathway plane”. Then, choose an appropriate “curved lead pathway” radius to calculate the equation of a circle on the “lead pathway plane”, which passes through both targets. This is the theoretical “curved lead pathway”. If the lead is delivered into the brain along the “curved lead pathway”, it can pass through both targets in sequence.

### Subjects and the designs of auxiliary devices

The accessory device to be used for human/primate is a new type of stereotaxic system, which secures the “curved lead pathway” via fixing two (or more) points on the pathway. Its application will be discussed in details in the Results section with the accompanying design schematics. A volunteer was scanned with 3.0 T MRI (Siemens Verio 3.0 T, Siemens AG, Erlangen, Germany). And the “curved lead pathways” of different combinations of targets were demonstrated on the head MR images. The study protocol was designed in accordance with guidelines outlined in the Declaration of Helsinki and approved by the Ethics Committee of the First Affiliated Hospital of Fujian Medical University (Fujian, China), and written informed consent was obtained from this volunteer.

The auxiliary device to be used for rat/mouse is different from a typical stereotaxic system, as it should to be used together with an animal stereotaxic frame and the stereotaxic coordinate atlas of rat/mouse brain. Procedures of making and using such an auxiliary device are explained in details with the design schematics in the Results section. Adult male Sprague-Dawley rats were housed individually under a 12-h reversed light/dark cycle, with no restrains in food and water access. Experimental protocols followed the guidelines of the National Institute of Health (NIH Publications No. 80–23, revised in 1996) and were approved and regulated by Institutional Animal Care and Use Committee of Fujian Medical University.

### Leads implantation

Twenty adult male Sprague-Dawley rats weighing 280–300 g were randomly assigned to four groups: for each rat in Group A (n = 5), two straight leads were implanted: one into the right subthalamic nucleus (STN, from bregma: 3.48 mm posterior, 2.6 mm lateral, and 8.0 mm ventral) and the other into right GP (from bregma: 1.08 mm posterior, 3.2 mm lateral, and 6.0 mm ventral) respectively; for each rat in Group B (n = 5), a cvrved lead was implanted into the right STN and GP; for each rat in Group C (n = 5), two straight leads, each into one STN were implanted; and for each rat in Group D (n = 5), a cvrved lead was implanted into the bilateral STN simultaneously. Because the curved lead is not yet commercially available, straight titanium (Ti) wires (with diameter of 0.4 mm; BaoTi Group Ltd, Baoji, China) and curved Ti wires (with diameter of 0.4 mm and arc radius of 9.2 mm) were used in place of the leads to evaluate the accuracy and reproducibility of curved lead implantation and the side effects caused by such a method. The coordinates of the targets in rat brain were determined with *The Rat Brain in Stereotaxic Coordinates of Paxinos and Watson (Sixth edition, 2007*)^38^. The Paxinos and Watson atlas was suitable for male Sprague-Dawledy rats with an average weight of 290 g used in this study^38^,^39^,^40^,^41^. Since there are several radii available for the “curved lead pathway”, and the bilateral STN occupy certain volume, there are more than one “curved lead pathways” that could pass the bilateral STN simultaneously. The Paxinos and Watson atlas^38^,^40^ is used to design the “curved lead pathway” that could minimize the impact of lead implantation to the surrounding brain tissue. For example, for rats in Group D (a lead pathway targeting bilateral STN and crossing the midline), a “curved path way” with 9.2 mm arc radius was selected. When the designed “curved lead pathway” crosses the midline, it passed through the posterior hypothalamus (a potential target for DBS to ameliorate akinesia in rat refs 42 and 43) and the upper edge of third ventricle, which potentially minimizes impacts to the important surrounding brain tissues: the curved lead avoids the dorsal part of posterior hypothalamic area above it, and the dorsomedial hypothalamic nucleus below it.

All rats were anesthetized by intraperitoneal injection of 40 mg/kg sodium pentobarbital. After anesthesia, the rats were mounted in a stereotaxic frame (RWD stereotaxic frame 68511, RWD Life Science Co., Ltd, Shenzhen, China), and straight Ti wires or curved Ti wires were implanted into the bilateral STN or right STN and GP. Dental cement was used to fasten the lead in position. All rats were given buprenorphine (0.12 g/kg), penicillin (80 g/kg), and saline (10 ml/kg), administered subcutaneously. Buprenorphine was also given every 12 h for 48 h postoperative for pain management.

### Morris water maze test

From the third to seventh day post-operation, the Morris water maze (MWM) test was used to evaluate spatial learning for the rats, following procedures described by Vorhees and Williams^44^. Briefly, a white pool (180 cm diameter, 50 cm deep) was filled with water to 30 cm high. Water temperature was maintained at 24 ± 1 °C. The target platform (a round plastic platform 12 cm in diameter) was positioned in the middle of the Northwestern quadrant and 1 cm below the water surface. Several highly visible maze cues were located in and around the pool. During acquisition trials, rats were randomized to one of four starting quadrants and given 1 min to find, climb, and remain on the platform for 5 s. Rats were then placed under a lamp to dry before their next run. Rats that failed to reach the platform within 1 min were guided to the platform and remained on it for 10 s. Four trials were conducted per day with a 15-min break between trials. The time until the rat climbed the platform (escape latency) was obtained using an automated video tracking system (AniLab Software & Instruments Co., Ningbo, China) for 4 consecutive days. The day after the acquisition phase, a probe trial was conducted and each rat was given 2 min to explore the pool. The time spent in each of four quadrants was measured.

### Verification of lead placement

After the MWM behavioral test, the rats were deeply anesthetized with sodium pentobarbital, and transcardially perfused with saline, then 4% polyformalin solution. Rat brains were obtained, sectioned, and stained with hematoxylin-eosin (H&E) to verify the lead placements. The locations of lead placement as well as potential lead-induced neuronal damages were assessed by an investigator who was unaware of the animals’ behavioral responses.

### The automated calculation algorithm

Several parameters, including the equation of the “curved lead pathway”, are needed for performing this implantation method. To simplify the calculation of the required parameters, Excel (Excel 2003, Microsoft Co., Richmond, Washington, USA) was used to program the automatic calculation algorithm.

### Statistics

The experimental data are presented as mean ± standard error of the mean (SEM). The data were statistically assessed with one-way ANOVA test followed by Tukey’s test for multiple post-hoc comparisons using SPSS 17.0 (SPSS Inc., Chicago, IL, USA). Statistical significance was defined as P < 0.05.

## Results

### Part one. Theoretical application of the “curved lead pathway” method on human

#### Stereotaxic system for implanting a curved lead

**Figure 1:**
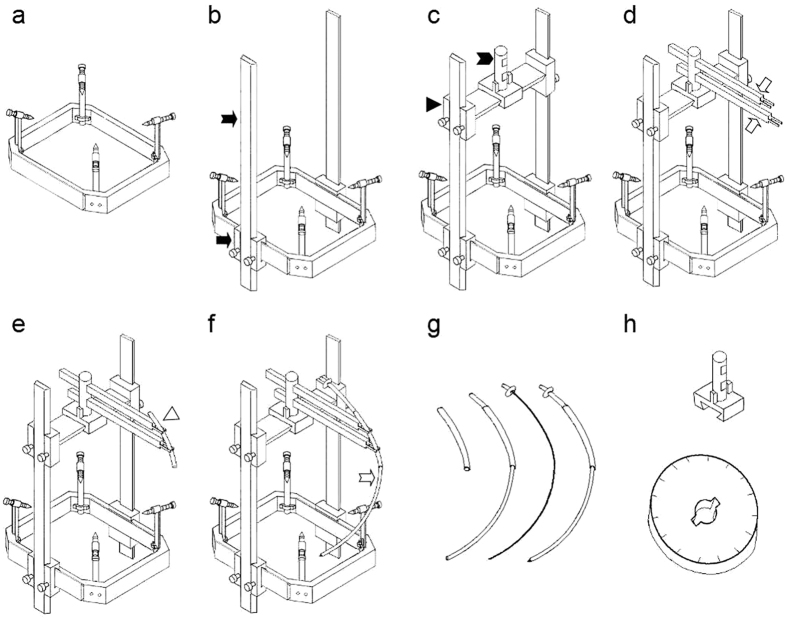
The proposed stereotaxic system for implanting a curved lead to human/primates. (**a**) The frame, including the bottom frame, supporting poles and skull screws. (**b**) The adaptor (black arrow) and vertical plank (black dovetail arrow) are added to the frame in figure a. (**c**) The horizontal plank (black triangle) and positioning pole (black dovetail arrow head) are added to the system in figure b. (**d**) The positioning poles (hollow arrow) are added to the system in figure c. (**e**) Positioning sheath (hollow triangle) is added to the system in figure d. (**f**) Trocar within the guiding sheath (hollow dovetail arrow) is added to the system in figure e. (**g**) From left to right: positioning sheath, positioning sheath with the guiding sheath passes through, and the trocar inserted in the guiding sheath. (**h**) From top to bottom: positioning base and the deflection angle measurement device.

A new type of stereotaxic system is needed to ensure that a single curved lead can reach at any two selected intracranial targets. As indicated in Fig. 1, the new stereotaxic system includes:

Frame: it is similar to the frame of the commonly used stereotaxic systems (such as that from Leksell), including the bottom frame (an octagonal frame), supporting poles, and skull screws (Fig. 1a).

X, Y, Z axes scale: the X, Y, Z axes are orthogonal to each other. X-axis scale is established on the horizontal plank (Fig. 1c), Y-axis scale is established on both sides of the frame (Fig. 1a), and Z-axis scale is established on the vertical plank (Fig. 1b).

Positioning poles, positioning base, and the deflection angle measurement device: they are used to determine the “lead pathway plane” and the “curved lead pathway”. The positioning poles are inserted into the positioning base through the two openings, and can be pushed forward or backward. The positioning sheath can be fixed to the tip of the positioning pole through rings, clipping, or fitting. The positioning base can be rotated to the calculated deflection angle with the help of the deflection angle measurement device (Fig. 1h).

Positioning sheath and guiding sheath: they are both curved hollow tubes used for guiding the lead. During lead implantation, the positioning sheath secures the guiding sheath, which guides the curved lead (Fig. 1g). For different pairs of targets, it is possible that the “curved lead pathways” might have different radii. Thus, several pairs of positioning and guiding sheaths with different arc radii were needed. Comparing between different pairs, the same type of sheaths should have the same inner and outer diameters.

#### The coordinates of the targets

The coordinates of targets in the 3D coordinate system of the stereotaxic system (CTiSS) are needed to calculate the “curved lead pathway”. CT/MRI images can be used to directly measure the CTiSS, if the targets are easily identifiable on these images. If the targets are hardly identifiable on these images, CTiSS can be obtained using the spatial relation between the targets and the mid-point of the anterior commissure- posterior commissure (AC-PC) line. We assign the deeper target to target 1 (X_1_, Y_1_, Z_1_), and the shallower target to target 2 (X_2_, Y_2_, Z_2_). The 3D coordinate system of the positioning frame system defines right as the positive X direction, forward as the positive Y direction, and upward as the positive Z direction.

#### Algorithm to calculate the necessary parameters

Excel was used to program the algorithm for calculating the parameters (Supplementary Table S1). To calculate the parameters, copy the contents in Supplementary Table S1 to the corresponding cells in the Excel sheet and fill in the needed information. Based on the equation of “curved lead pathway” (an equation of a circle), there will be two sets of results for l_1_ and l_2_, the algorithm will show the set that may have a shorter length for the part of the curved lead implanted into the brain.

#### Steps of curved lead implantation

The steps include:

1. Acquire the coordinates of the two targets: obtain the CTiSS and adjust the horizontal and vertical planks of the new stereotaxic system, such that target 1 (the deeper target) is at the “origin” of the system.
2. Establish the “lead pathway plane”: the “lead pathway plane” is orthogonal to the bottom frame and contains both targets. Input the coordinates of the two targets into the Excel algorithm to obtain the “deflection angle” and the “deflection direction”. Rotate the positioning pole with the initial forward orientation towards “counter-clockwise” or “clockwise” for the degree of the “deflection angle” (in Excel algorithm, the “deflection direction” will be shown as the “←” or “→”), then both positioning poles fall into the “lead pathway plane”.
3. Identify the “curved lead pathway”: select an appropriate curved lead pathway radius (r), measure h_1_ and h_2_, and input r, h_1_, h_2_ into the algorithm to obtain l_1_ and l_2_ (Supplementary Table S1). Pass the two positioning poles through the holes on the positioning base at lengths l_1_ and l_2_ (l_1_ is the length of the higher pole) (Fig. 1d), such that the tips of positioning poles reach two points (h_1_,l_1_) and (h_2_,l_2_) on the “curved lead pathway” respectively. Secure the positioning sheath at the tip of the poles, then the sheath will overlap with the “curved lead pathway” (Fig. 1e).
4. Implant the curved lead: based on the calculated penetration depth, pass the guiding sheath through the positioning sheath and push it into the brain tissue. The curved guiding sheath moves along with the “curved lead pathway” and passes one target after another (Fig. 1f). Replace the trocar in guiding sheath with the curved lead, and then retrieve the guiding sheath.

### Design the “curved lead pathway” on MRI

**Figure 2:**
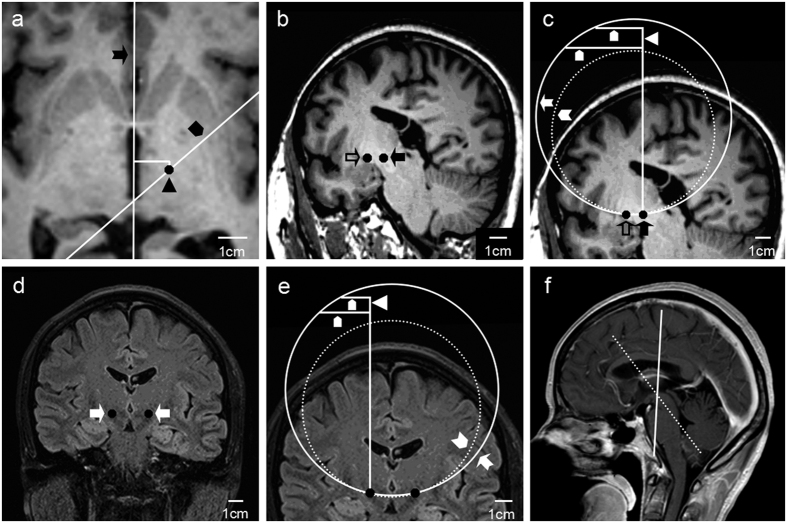
Design the “curved lead pathway” on MR images. (**a–c**) The “curved lead pathways” passing through the right STN and right GPi. a. The axial image of the AC-PC plane. The lead pathway plane (black pentagon) passes through the point (12, −3, 0) (black triangle) and forms a 49.4 degree angle with the medium sagittal plan (black dovetail arrow). (**b**) Reconstruct the lead path plane on the MRI-workstation of the device (Siemens Verio 3.0 T, Siemens AG, Erlangen, Germany). The lead pathway plane is orthogonal to the AC-PC plane, and passes through the right GPi (hollow arrow) and STN (black arrow). (**c**) Identify the “curved lead pathway” on the “lead pathway plane”. When r = 5 cm, “curved lead pathway” is indicated by the white dovetail arrow head. When r = 6 cm, the “curved lead pathway” is indicated by the white dovetail arrow, and the estimated locations of the positioning base and poles are indicated by the white triangle and white pentagon, respectively. (**d–f**) The “curved lead pathways” passing through the bilateral STN. (**d**) The lead pathway plane, which is orthogonal to the sagittal plane and perpendicular to the AC-PC plane, passes through the bilateral STN (white arrow). (**e**) Identify the “curved lead pathway” on the “lead pathway plane”. When r = 5 cm, the “lead pathway” is indicated by the white dovetail arrow head. When r = 6, “curved lead pathway” is indicated by the white dovetail arrow, and the estimated location of the positioning base and poles are indicated by the white triangle and white pentagon, respectively. The lead pathway passes through the parietal lobe. (**f**) Adjust the safe entry point of the lead by adjusting the location of the positioning frame. When the plane of the bottom frame (the XOY plane) is parallel to the AC-PC plane, the “lead pathway plane” is indicated by the solid white line, and the lead entry point is located at the parietal bone. If the bottom frame is leaned forward for 30–40°, the approximate position of the “lead pathway plane” is indicated by the dash line, and the lead entry point is located at the frontal bone.

A volunteer was scanned with 3.0 T MRI, the bottom frame (XOY plane) was parallel to the AC-PC plane. If the STN and GPi on the right side were selected as targets, the coordinates of targets 1 and 2 are (12, −3, −5) and (19, 3, −5) in mm (the origin of coordinate system is the mid-point of AC-PC line), and the “curved lead pathway” is demonstrated in Fig. 2a–c. If the bilateral STN were selected, the coordinates of targets 1 and 2 are (12, −3, −5) and (−12, −3, −5) in mm, and the “curved lead pathway” is demonstrated in Fig. 2d–f.

### Part two. Application of the “curved lead pathway” method on rat

#### Obtain the coordinates of the targets

To ensure the accuracy of implantation, the auxiliary device should be used together with the stereotaxic atlas of rat/mouse brain. To make the auxiliary device the coordinates of the targets and the “curved lead pathway” should be identified first.

The intracranial coordinates of rat/mouse can be referred to the stereotaxic atlases that are based on the horizontal plane of the skull^38^,^40^,^45^. In these atlases:1. X represents the lateral distance from the mid-line (in the Excel algorithm, we choose right as the positive direction); 2. Y represents the frontal distance from the bregma; 3. Z represents the vertical distance from the “0 horizontal plane”. All units are in mm.

#### Algorithm to calculate the necessary parameters

Excel was used to program the automated algorithm (Supplementary Table S2). The necessary parameters can be calculated by copying the contents in Supplementary Table S2 to the corresponding cells in an Excel sheet and filling in the needed information.

#### Auxiliary device for implanting a curved lead

**Figure 3:**
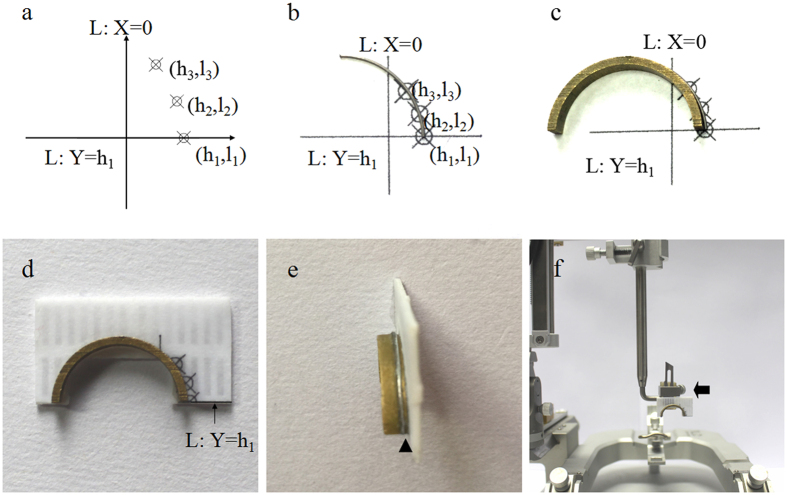
Procedures of making the auxiliary device for rat/mouse. (**a**) Three points ((l_1_, h_1_), (l_2_, h_2_) and (l_3_, h_3_)) on the “curved lead pathway”, line X = 0 and line Y = h_1_ are drawn with AutoCAD software 2009(Autodesk Inc., San Francisco, California, USA) and printed. Line Y = h_1_ represents the 0 horizontal plane. (**b**) Place the curved lead (represented by a curved metal wire) horizontally on the drawing such that it overlaps with the “curved lead pathway”. (**c**) Place the carrier such that it completely touches the curved lead, and glue the carrier on the drawing such that its front end reaches line Y = h_1_. (**d**) Cut the part of the drawing with the glued carrier, and glue a piece of plastic to its back for reinforcement. The drawing and the piece of plastic are also used as one side of the “lead channel”. (**e**) The other side of the “lead channel” is made on the surface of the carrier using metal wire (black triangle), the width of the “lead channel” depends on the diameter of the curved lead. (**f**) Glue the handle (black arrow), through which the auxiliary tool is connected to the stereotaxic frame.

An auxiliary device is needed to implant a curved lead into the brain of rat/mice. The procedure of making the auxiliary device is demonstrated in Fig. 3. With the following steps: 1. Select two targets, and choose a curved lead with an appropriate arc radius. The radius of the curved lead is equal to that of the “curved lead pathway”. 2. Calculate the necessary parameters: input coordinates of the deeper target (X_1_, Y_1_, Z_1_) and shallower target (X_2_, Y_2_, Z_2_) and the radius of the “curved lead pathway” to obtain (l_1_, h_1_), (l_2_, h_2_), and (l_3_, h_3_). 3. Make a carrier for the “curved lead pathway”: choose a piece of curved material with the same curvature as the curved lead (for example, this can be achieved by cutting different sized metal capillaries into half or 1/4 circles), and follow steps in Fig. 3a–c to make the carrier for the “lead pathway”. 4. Make the “lead channel” on the carrier following the steps on Fig. 3d–e. This channel restrains the movements of the curved lead. 5. Make the handle of the auxiliary device: glue the handle to the carrier for fixing to the stereotaxic apparatus (Fig. 3f). The intersection of the “lead channel” and the line Y = h_1_ is an important point (Fig. 3d) used to locate the lead entry point on the “0 horizontal plane”. The portion of line Y = h_1_ on the schematic will be used to adjust the relative position between the bottom of auxiliary device and the horizontal plane.

#### Steps of curved lead implantation

The steps are: 1. Secure the auxiliary device on the stereotaxic frame: when securing, ensure that line Y = h_1_ on the auxiliary device is parallel to the horizontal platform of the stereotaxic apparatus. 2. Adjust the position of the auxiliary device: rotate the operation arm of the stereotaxic frame for animal such that the “lead channel” forms the “deflection angle” with the Y axis. In this step, it would be helpful to print a picture with the “deflection angle” and place it on the horizontal platform of the stereotaxic apparatus. 3. Identify the “0 horizontal plane”: secure the rat/mouse on the stereotaxic frame, adjust the incisor bar and the ear bar such that the bregma and lambda are on the same horizontal plane. 4. Adjust the position of “lead channel”: move the auxiliary device such that the intersection of the lead channel and the line Y = h_1_ reaches the bregma; zero the stereotaxic frame; and then move the intersection to the lead entry point (X_a_, Y_a_). With this procedure, the “lead channel” will overlap with the “curved lead pathway”. 5. Implant the curved lead along the “lead channel”, the implantation length is calculated by the Excel algorithm.

#### Curved lead implantation in rats

**Figure 4:**
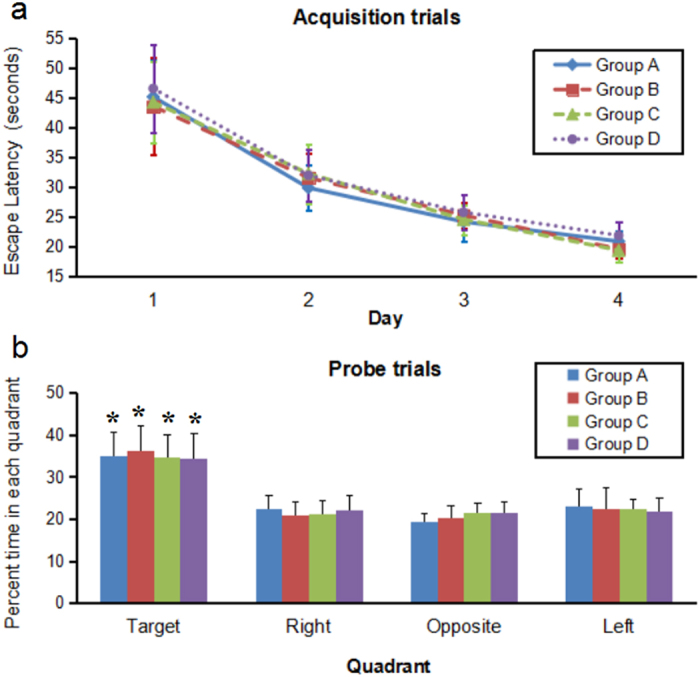
Performance in the MWM tests. All rats underwent the MWM tests had four acquisition trials daily for four days and a probe trial. (**a**) Acquisition trials. No difference between the straight lead groups and the curved lead groups was observed. As acquisition training progressed, all groups exhibited significant decrease in the latency required to find the platform. (**b**) Probe trial. The probe trial showed that all groups of rats spent a significant amount of time searching in the quadrant where the platform was previously located (*P < 0.001, respectively). And there was no significant difference between the four groups. The results were expressed as mean ± SEM. *P* values were determined using ANOVA. MWM: Morris water maze.

**Figure 5:**
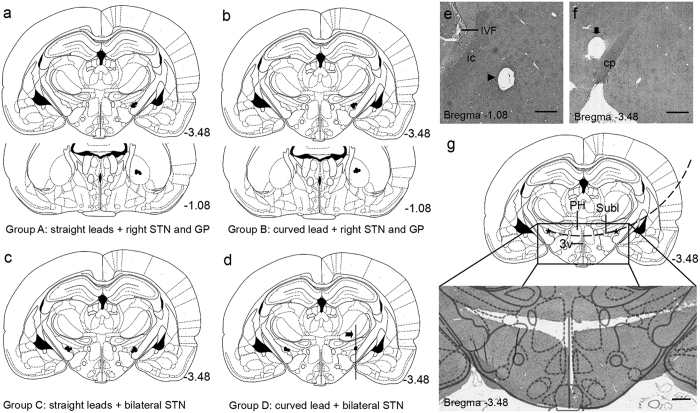
Location of the lead placement and the “curved lead pathway”. (**a–d**) Schematic illustrations of the leads localized in the selected targets. The location of the lead tip of the straight lead was illustrated (**a** and **c**). And the location of the lead tip and the point of the curved lead passing through the coronal section 1.08 mm posterior to the bregma (**b**) or the marker line (2.6 mm mediolateral, black dovetail arrow, (**d**) were also illustrated. Leads were located within the boundaries of the selected targets in all implanted rats. (**e,f**) The coronal sections of the brain of a rat in Group C. Two holes were observed at the right GP (figure e, black triangle) and right STN (**f**) black arrow) respectively, which were caused by an implanted curved lead. (**g**) The “curved lead pathway” that passing through bilateral STN (black dashed line) could be designed on the coronal section at 3.48 mm posterior to the bregma. It can be observed on the HE stained coronal brain section that the implanted lead (black line) passes the bilateral STN. “Curved lead pathway” crosses the midline, passes through posterior hypothalamus (**a**) potential target for DBS to ameliorate akinesia in rat^42^,^43^) and the upper edge of third ventricle. IVF: interventricular foramen; ic: internal capsule; cp: cerebral peduncle; 3 V: the third ventricle; PH: posterior hypothalamus; Subl: subincertal nucleus. The scale bars represent 500 micrometers. The number at the bottom of each panel corresponds to the distance from bregma in mm, according to the Paxinos and Watson atlas^38^. Figures a-d and f were modified from *The Rat Brain in Stereotaxic Coordinates of Paxinos and Watson*^40^ (reproduced with permission).

For rats in Groups A and B, the right STN and GP were selected as the targeted nuclei. For rats in Groups C and D, the bilateral STN were selected as target nuclei. Two straight leads were implanted in each rat in Groups A and C, and a curved lead was implanted in each rat in Groups B and D. The effects of curved lead implantation on spatial learning and memory were assessed using MWM tasks. There were no differences between the four groups on the performance during either the acquisition or the probe tests (Fig. 4). At the seventh day post-implantation, the rat brains were obtained and processed for histological analysis. The brain sections stained with H&E staining showed that all of the straight leads and the curved leads were implanted accuratly (Fig. 5).

However, although the curved leads were located within the boundaries of the STN or GP in all implanted rats, most of the curved leads implanted to the rats of Group D seemed to be a litter higher (with the variations of ≤0.1 mm) than the planned positions.

## Discussion

This paper proposes for the first time a method that enables a single lead to reach any two targets of the brain. The new kind of stereotaxic system and auxiliary device needed for curved lead implatation were designed and/or fabricated (Figs 1 and 3), and the Excel algorithm needed to calculate the necessary parameters was designed (Supplementary Table S1 and Supplementary Table S2). In the current study, the potential pathways for curved lead implantation that target the ipsilateral STN-GPi or bilateral STN were designed and drawn on MRI (Fig. 2). It was also demonstrated in the rat experiment that with this “curved lead pathway” method, a curved Ti wire can reach either the ipsilateral GP-STN or bilateral STN. Conventionally, the “straight lead pathway” method is used for lead implantation. Under most circumstances, multi-target simulation/recording can only be achieved via increased number of leads^18^,^19^,^20^,^21^,^22^,^23^,^24^,^27^,^31^. In this case, the “curved lead pathway” method may complement the current “straight lead pathway” method, make lead implantation for multiple targets more convenient, and expand experimental techniques of brain function research.

To realize such an implantation method in human, we designed the stereotaxic system needed for human/primate use. Several researchers^22^,^23^,^24^,^46^ have reported the clinical use of multi-target stimulation. For example, Peppe *et al*.^24^ performed STN-DBS and pedunculopontine nucleus (PPN)-DBS spontaneously, and found that STN-+PPN-DBS might lead to better results compared to STN-DBS when it comes to treating gait irregularity. The multi-target stimulation aimed at treating symptoms with different pathogenetic mechanisms related to different nuclei, might be a promising research direction in the next couple of years^47^. Furthermore, although STN-DBS and GPi-DBS lead to similar treatment results for Parkinson’s disease^48^,^49^,^50^, some researchers^51^,^52^ reported that when GPi-DBS was performed on patients with decreased treatment efficiency by STN-DBS, the symptoms can be alleviated again. To prevent failure of DBS treatment due to the reduced efficiency of single target stimulation, a potential solution is to implant a curved lead into the STN and GPi spontaneously.

To enable the lead implantation in rat/mice, the auxiliary device was also fabricated and tested. Results showed that the auxiliary device could guide the curved Ti wires to reach the two selected targets with high accuracy (Fig. 5g), and the potential lead-induced neuronal mechanical damage was not found. However, although the curved leads were located within the boundaries of the STN or GP in all implanted rats, most of the curved leads implanted to the rats in Group D seemed to be a litter higher (with the variations of ≤0.1 mm) than the positions of the selected points. This might be caused by the brain sag and pneumocephalus during the implantation process. Guo *et al*.^53^ reported that the short-term side effects of lead implantation include mild confusion, central fever and recent memory excalation, all of which were transient and reversible. The effects of curved lead implantation on spatial learning and memory have been assessed using MWM tasks. There were no differences between curved lead or straight lead implanted groups on the performance during either the acquisition phase or the probe test. Moreover, it might be important to note that although non-motor side effects of lead implantation were not explicitly examined, no overt changes in behavior were observed in the rats with curved lead implanted. This preliminary study indicated that curved lead implantation is relatively safe with an appropriately designed curved lead pathway. As the research on brain function moves forward, more and more researchers^26^,^27^,^31^,^32^,^33^ worked on stimulating or recording multiple intracranial targets. The “curved lead pathway” method might contribute to the further development of electrophysiological techniques.

There are three critical steps in curved DBS lead implantation in rat: first, design an appropriate curved lead pathway. The designed curved lead pathway should have proper entry point to avoid major blood vessels and sulci. This can be achieved by combining the atlas, MR images, and the automated Excel calculation algorithm. For example, enhanced CT/MRI can be used to better identify and void vessels on the “curved lead pathway”. When measuring the coordinates of the targets, a 3D coordinate system was established. In this 3D coordinate system, the point (h_1_,l_1_) from the Excel algorithm is on the “curved pathway”. Using the formulas used for calculating (h_1_,l_1_), the points of “curved lead pathway” on axial planes (these planes are parallel to the XOY plane) can be identified: h_1_ represents the height difference between the specific axial plane and the deeper target, and l_1_ can be calculated using the Excel algorithm. The coordinates of the intersection between the “curved lead pathway” and the axial plane is (X_1_ +/− l_1_*sin(deflection angle), Y_1_ +/− l_1_*cos(deflection angle)).The calculated X value should be between X_1_ and X_2_, and the Y value should be between Y_1_ and Y_2_. This intersection is observed to examine if it overlaps with any major blood vessels or sulci. If overlap happened, the XOY plane or the radius of the “curved lead pathway” can be adjusted to re-design the “curved lead pathway”. For example, in Fig. 2f, when the plane of the bottom frame (the XOY plane) is parallel to the AC-PC plane, the “lead pathway plane” is indicated by the solid white line, and the lead entry point is on the parietal bone. If the bottom frame is leaned forward for 30–40°, then the approximate position of the “lead pathway plane” is indicated by the dash line, and the lead entry point is at the frontal bone.

Second, make the auxiliary device. The procedure of making the auxiliary device is demonstrated in Fig. 3. Results showed that the auxiliary device could guide the curved lead to the two selected intracranial targets with high accuracy. However, the accessory device in the current study was still made manually, which could lead to inaccuracy when the fabrication process was repeated. We suggest that researchers could follow the method and designed schematics proposed here, and use more automated methods, such as 3D printing to fabricate the auxiliary device.

Third, choose an appropriate curved lead. In this study, we used curved Ti wires instead of actual curved DBS leads since they are not yet commercially available. The leads currently used cannot meet the requirements for “curved lead pathway” method to the full extend. Since curved lead needs to penetrate through brain of rat, it should be made from hard material. Currently, the multiple-channel lead already enables stimulation of two targets with optimal parameters, by adjusting the stimulating parameters of each channel. Therefore, in designing the actual curved lead, we plan to have a short touch point 1 at the tip of the lead, and a long touch point 2 behind the tip of the lead, which is covered by an insulation layer. In actual use, the distance between the two touch points on the lead can be calculated. Based on this distance, the insulation layer can be removed on the corresponding location of touch point 2 to reveal a small simulating point. Following this method, the lead has a wide range of applications, and can be used for different combinations of the target.

Moreover, the DBS lead used in clinical practice cannot completely meet the requirements for the “curved lead pathway” method. For example, the sheath has certain tension, when implanted curved, the shape of lead might change after the surgery. Therefore, this implantation method calls for new kinds of lead. Potential solutions may include: 1. the curved lead can be manufactured from hard material and customized for different combinations of targets, as hard material is more resistant to change in shape. When removal of lead is needed, a curved positioning sheath can be used to ease the retrieval process. 2. Select material with even smaller tension to fabricate the lead sheath. A soft curved lead can be used for different combinations of the targets, as long as the lead-contact distance is appropriate. And it is less likely for the soft lead to cause brain tissue damage when the brain is moving at changing speeds. It is believed that the lead fabrication process will develop further as the result of improved lead implantation method.

There are some limitations in this study. First, although a new stereotaxic system was designed, and the “curved lead pathway” for two combinations of targets was demonstrated on images, the human part in Results is theoretical. Our next challenge is to manufacture a stereotaxic system for implanting a curved lead to two intracranial targets with high accuracy. Second, the rat experiments showed that by appropriately design the “curved lead pathway”, no additional side effects were brought by the curved lead compared to straight lead. However, the sample size in this study is relatively small, and only the STN and GP were selected as the targets. Further work is required to determine how safe and accurate this method is for different combinations of targets, evaluate potential side effects, and establish a more complete procedure for “curved lead pathway” design. Third, in this study, we used adult male Sprague-Dawley rats weighing 280–300 g, but the Paxinos and Watson atlas is developed from study of adult male Wistar rats with weights ranging from 270 to 310 g. Althought the Paxinos and Watson atlas is also suitable for the male Sprague Dawley rats with the same weight^38^,^39^,^40^,^41^, there might be a few of anatomic differences between the Sprague-Dawley rat brain and the Paxinos and Watson atlas.

## Additional Information

**How to cite this article**: Ding, C.-Y. *et al*. The “curved lead pathway” method to enable a single lead to reach any two intracranial targets. *Sci. Rep.* **7**, 40533; doi: 10.1038/srep40533 (2017).

**Publisher's note:** Springer Nature remains neutral with regard to jurisdictional claims in published maps and institutional affiliations.

## Supplementary Material

Supplementary Tables
