# Supplementary material for: The “curved lead pathway” method to enable a single lead to reach any two intracranial targets
Source: Sci Rep. 2017 Jan 11;7:40533. doi: 10.1038/srep40533 (PMC5225435; doi:10.1038/srep40533)
Supplement: Supplementary Tables [file srep40533-s1.doc]

**The “curved lead pathway” method to enable a single lead to reach any two intracranial targets**

Chen-Yu Ding, Liang-hong Yu, Yuan-Xiang Lin, Fan Chen, Zhang-Ya Lin, De-Zhi Kang*

Department of Neurosurgery, The First Affiliated Hospital of Fujian Medical University, Fuzhou, China.

*Corresponding author:

Dezhi Kang, Department of Neurosurgery, The First Affiliated Hospital of Fujian Medical University, Fuzhou, China.

E-mail: kdz99988@vip.sina.com

**Running title:** Method to enable a lead to reach two targets

**Number of supplementary tables:** 2

| Supplementary Table S1. Excel algorithm for calculating the necessary parameters of “curved lead pathway” method used in human/primate. | | | | |  |
| --- | --- | --- | --- | --- | --- |
| Column | Row 1 | Row 2 | Row 3 | |  |
| A | Basic information | Name or No | Fill in | | |
| B |  | The targeted area | Fill in | | |
| C | The Deeper Target | X_1_ | Fill in after measurement | |  |
| D |  | Y_1_ | Fill in after measurement |  | |
| E |  | Z_1_ | Fill in after measurement |  | |
| F | The Shallower Target | X_2_ | Fill in after measurement | |  |
| G |  | Y_2_ | Fill in after measurement |  | |
| H |  | Z_2_ | Fill in after measurement |  | |
| I | The selected radius | r | Fill in after selection | |  |
| J | Positioning pole 1 | h_1_* | Fill in after measurement | | |
| K |  | l_1_* | =IF(OR(T3="→",T3="↗",T3="↑",T3="↖"),R3+SQRT(I3^2-(S3-J3)^2),R3-SQRT(I3^2-(S3-J3)^2)) | | |
| L | Positioning pole 2 | h_2_* | Fill in after measurement | |  |
| M |  | l_2_* | =IF(OR(T3="→",T3="↗",T3="↑",T3="↖"),R3+SQRT(I3^2-(S3-L3)^2),R3-SQRT(I3^2-(S3-L3)^2)) |  | |
| N | Lead pathway plane | Deflection angle† | =ACOS(ABS(D3-G3)/SQRT((C3-F3)^2+(D3-G3)^2))/PI()*180 | |  |
| O |  | Deflection direction‡ | =IF(C3=F3,"↑",IF(D3=G3,IF(C3>F3,"←","→"),IF(C3>F3,IF(D3>G3,"→","←"),IF(D3>G3,"←","→")))) |  | |
| P |  | X'§ | =IF(OR(T3="→",T3="↗",T3="↑",T3="↖"),1,-1)*SQRT((C3-F3)^2+(D3-G3)^2) |  | |
| Q |  | Y'§ | =ABS(E3-H3) |  | |
| R | The center of the lead pathway circle | Xo¶ | =Q3^2/(2*P3)+P3/2-Q3*S3/P3 | |  |
| S |  | Yo¶ | =(((P3^2*Q3+Q3^3)/P3^2)+SQRT((-(P3^2*Q3+Q3^3)/P3^2)^2-4*((Q3/P3)^2+1)*(((P3^2+Q3^2)/(2*P3))^2-I3^2)))/(2*((Q3/P3)^2+1)) |  | |
| T | Direction of the arch bump of lead placement** | | =IF(C3=F3,IF(D3>G3,"↓","↑"),IF(D3=G3,IF(C3<F3,"→","←"),IF(C3>F3,IF(D3>G3,"↙","↖"),IF(D3>G3,"↘","↗")))) | |  |
| U | The arc length of the guiding sheath †† | | =2*PI()*I3*(2*ASIN(SQRT(J3^2+K3^2)/(2*I3))/PI()*180)/360 | |  |
| V | Arc distance between the two targets on the “curved lead pathway” ‡‡ | | =2*PI()*I3*(2*ASIN(SQRT(P3^2+Q3^2)/(2*I3))/PI()*180)/360 | |  |
| *(l_1_, h_1_) and (l_2_, h_2_): the coordinates of two points on the curved lead pathway. The “h” represents the relative height between the positioning pole and the deeper target, and the “l” represents the length of positioning pole after it passed through the mid-line of the positioning pin. †Deflection angle: the angle formed between the lead pathway plane and the sagittal plane. ‡Deflection angle: shown as “↑", “←" or "→", meaning that if the positioning pole, which is oriented towards the positive direction and on the sagittal plane that passes through the deeper target, is rotated “←” or “→” for the degree of “deflection angle” using the positioning base as the rotation axis, will fall onto the “lead pathway plane”. §(X', Y'): the coordinates of the shallower target on the “lead pathway plane”. ¶(Xo, Yo): the coordinates of the center of the circle (curved lead pathway) on the lead pathway plane. **Direction of arc bump: it represents the direction of arc bump of the curved lead pathway. The possible outcomes include “←”, “↖”, “↑”, “↗”, “→”, “↘”, “↓”, and “↙”. “↑” represents forward direction (with 0 degree deviation), “↗” represents forward direction with 45 degree deviation to the right, etc. ††The penetration depth of the guiding sheath: the arc length of the guiding sheath passes through the point (l_1_, h_1_) along the “curved lead pathway”. ‡‡The arc distance between the two targets on the “curved lead pathway”: this parameter is used to select the optimal lead with appropriate distance between lead-contacts. The units of input and output parameters are in mm. | | | | |  |

| Supplementary Table 2. Excel algorithm for calculating the necessary parameters of “curved lead pathway” method used in rat/mice. | | | |
| --- | --- | --- | --- |
| Column | Row 1 | Row 2 | Row 3 |
| A | Basic information | Name or No | Fill in |
| B |  | The targeted area | Fill in |
| C | The Deeper Target | X_1_ | Fill in after measurement |
| D |  | Y_1_ | Fill in after measurement |
| E |  | Z_1_ | Fill in after measurement |
| F | The Shallower Target | X_2_ | Fill in after measurement |
| G |  | Y_2_ | Fill in after measurement |
| H |  | Z_2_ | Fill in after measurement |
| I | The selected radius | r | Fill in after selection |
| J | The 0 horizontal plane* | h_1_† | =E3 |
| K |  | l_1_† | =IF(OR(V3="→",V3="↗",V3="↑",V3="↖"),T3+SQRT(I3^2-(U3-J3)^2),T3-SQRT(I3^2-(U3-J3)^2)) |
| L | 3mm  3mm above the 0 horizontal plane | h_2_† | =E3+3 |
| M |  | l_2_† | =IF(OR(V3="→",V3="↗",V3="↑",V3="↖"),T3+SQRT(I3^2-(U3-L3)^2),T3-SQRT(I3^2-(U3-L3)^2)) |
| N | 6mm  6mm above the 0 horizontal plane | h_3_† | =E3+6 |
| O |  | l_3_† | =IF(OR(V3="→",V3="↗",V3="↑",V3="↖"),T3+SQRT(I3^2-(U3-N3)^2),T3-SQRT(I3^2-(U3-N3)^2)) |
| P | Lead pathway plane | Deflection angle | =ACOS(ABS(D3-G3)/SQRT((C3-F3)^2+(D3-G3)^2))/PI()*180 |
| Q |  | Deflection direction | =IF(C3=F3,"↑",IF(D3=G3,IF(C3>F3,"←","→"),IF(C3>F3,IF(D3>G3,"→","←"),IF(D3>G3,"←","→")))) |
| R |  | X' | =IF(OR(V3="→",V3="↗",V3="↑",V3="↖"),1,-1)*SQRT((C3-F3)^2+(D3-G3)^2) |
| S |  | Y' | =ABS(E3-H3) |
| T | The center of the lead pathway circle | Xo | =S3^2/(2*R3)+R3/2-S3*U3/R3 |
| U |  | Yo | =(((R3^2*S3+S3^3)/R3^2)+SQRT((-(R3^2*S3+S3^3)/R3^2)^2-4*((S3/R3)^2+1)*(((R3^2+S3^2)/(2*R3))^2-I3^2)))/(2*((S3/R3)^2+1)) |
| V | Direction of the arch bump of lead placement | | =IF(C3=F3,IF(D3>G3,"↓","↑"),IF(D3=G3,IF(C3<F3,"→","←"),IF(C3>F3,IF(D3>G3,"↙","↖"),IF(D3>G3,"↘","↗")))) |
| W | Length for the implanted lead‡ | | =2*PI()*I3*(2*ASIN(SQRT(J3^2+K3^2)/(2*I3))/PI()*180)/360 |
| X | Arc distance between the two targets on the “curved lead pathway” | | =2*PI()*I3*(2*ASIN(SQRT(R3^2+S3^2)/(2*I3))/PI()*180)/360 |
| Y | The entry point of lead on the “0 horizontal plane” | Xa'¶ | =SIN(RADIANS(P3))*ABS(K3)*IF(OR(V3="→",V3="↗",V3="↘"),1,-1)+C3 |
| Z |  | Ya'¶ | =COS(RADIANS(P3))*ABS(K3)*IF(OR(V3="↗",V3="↖",V3="↑"),1,-1)+D3 |
| *0 horizontal plane: the horizontal plane that passes both the bregma and lambda. †(l_1_, h_1_), (l_2_, h_2_) and (l_3_, h_3_): the coordinates of three points on the “curved lead pathway”; ‡Length of the implanted lead: on the “curved lead pathway”, the arc length from point of entry on the “0 horizontal plane” to the deeper target. ¶(Xa,Ya): the coordinates of the intersection point between the “curved lead pathway” and the “0 horizontal plane”. The units of input and output parameters are in mm. | | | |
